# Effect of Microgravity on Endothelial Cell Function, Angiogenesis, and Vessel Remodeling During Wound Healing

**DOI:** 10.3389/fbioe.2021.720091

**Published:** 2021-09-22

**Authors:** Lucia Morbidelli, Shirley Genah, Francesca Cialdai

**Affiliations:** ^1^ Department of Life Sciences, University of Siena, Siena, Italy; ^2^ ASA Campus Joint Laboratory, ASA Research Division & Department of Experimental and Clinical Biomedical Sciences “Mario Serio”, University of Florence, Florence, Italy

**Keywords:** wound healing, angiogenesis, angiogenic factors, endothelial cells, microgravity, drugs, cell therapy, physical therapy

## Abstract

Wound healing is a complex phenomenon that involves different cell types with various functions, i.e., keratinocytes, fibroblasts, and endothelial cells, all influenced by the action of soluble mediators and rearrangement of the extracellular matrix (ECM). Physiological angiogenesis occurs in the granulation tissue during wound healing to allow oxygen and nutrient supply and waste product removal. Angiogenesis output comes from a balance between pro- and antiangiogenic factors, which is finely regulated in a spatial and time-dependent manner, in order to avoid insufficient or excessive nonreparative neovascularization. The understanding of the factors and mechanisms that control angiogenesis and their change following unloading conditions (in a real or simulated space environment) will allow to optimize the tissue response in case of traumatic injury or medical intervention. The potential countermeasures under development to optimize the reparative angiogenesis that contributes to tissue healing on Earth will be discussed in relation to their exploitability in space.

## Introduction

The skin is the largest organ by surface area in the human body. Its structure is highly organized with different cell types (epithelial, stromal, and endothelial cells-ECs), which finely cooperate in order to guarantee a constant functioning and structural homeostasis of the organ. Indeed, the skin is the first defensive barrier of our body that protects internal tissues from microbial infections, mechanical damages, UV radiations, dangerous substances, and high temperatures. Therefore, the maintenance of its integrity is fundamental for our survival and skin repair, following a mechanical or physical injury, implying very complex and delicate processes to recover its integrity and barrier function (Sorg et al., 2017; Rodrigues et al., 2019). Here we report the state-of-the art of tissue wound healing phases and factors, the role of angiogenesis and the potential pharmacological, cellular, and physical countermeasures acting on ECs. The aim of the article is to understand how to apply all these findings in the space environment as the one that astronauts face during long duration missions. Indeed during space travels the possibility for astronauts to hurt themselves during their routine activities is not excluded. The availability of countermeasures with verified effectiveness and safety in unloading conditions will guarantee the optimal tissue healing and health recovery in extreme environments without the urgency to rapidly return to Earth. The review article is based on the recent/most cited papers of PubMed and Scopus databases on the topic of reparative angiogenesis, wound healing, and related countermeasures.

## Wound Healing Phases

**FIGURE 1:**
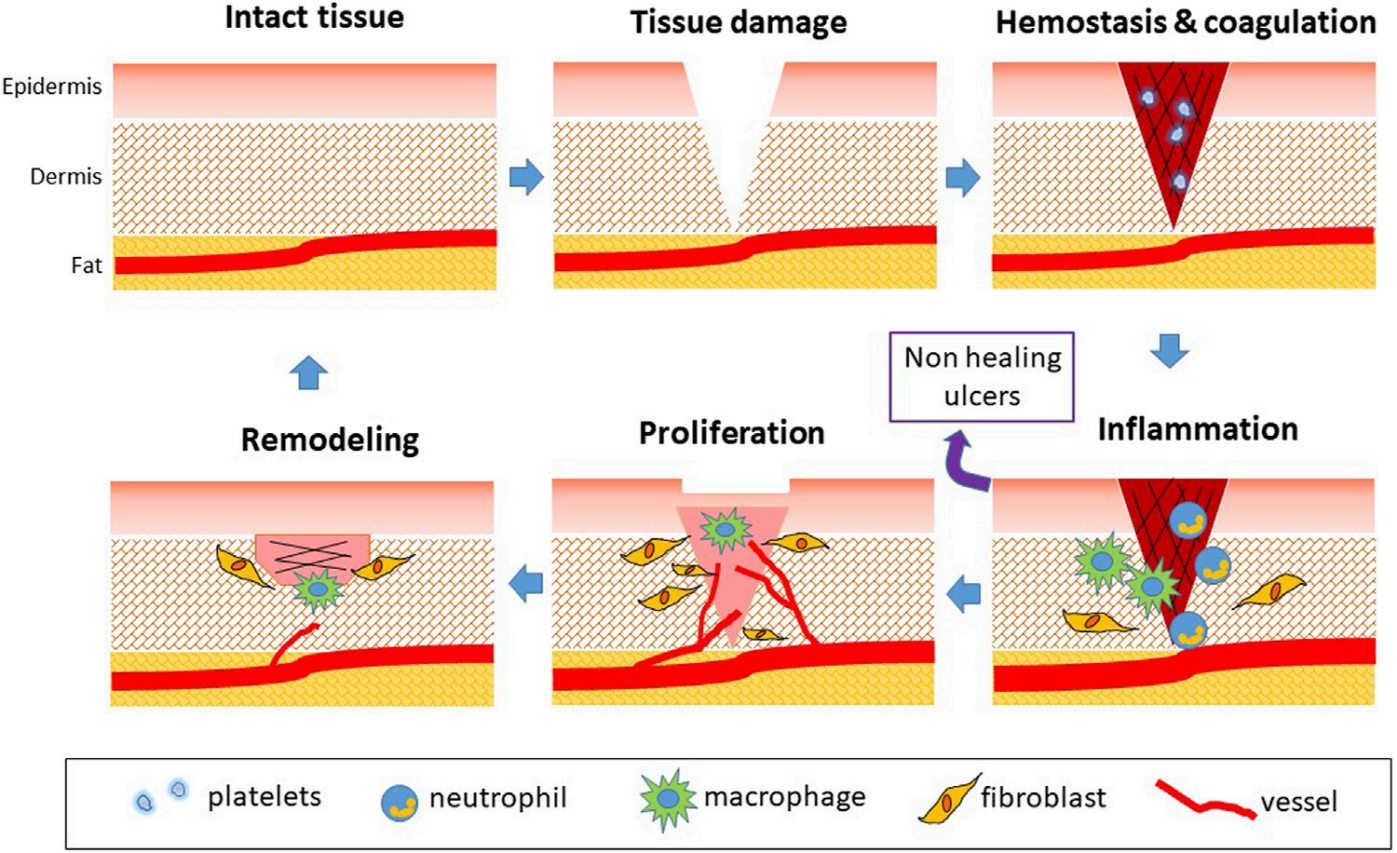
Phases of physiological wound healing. Following tissue injury, hemostasis occurs. Coagulation is responsible for fibrin clotting and cessation of blood flowing. Platelets activate and secrete PDGF, EGF, TGF-β, CXCL4, and PF4. The following phase is represented by inflammation, also induced by tissue debris and bacteria. Vessels dilate in a NO-dependent manner. Neutrophils (0–3 days) exert antimicrobial activity, secreting MMPs and cytokines as TNF-α and IL-1/4/6. Monocytes are attracted (1–7 days) and transform in M1 macrophages involved in host defense with phagocytic activity. ROS are generated to eliminate bacteria. Then, M2 phenotype occurs with the production of growth factors involved in tissue repair. The proliferation phase is characterized by angiogenesis occurrence due to release of VEGF and FGF, EC proliferation and migration and fibroblast proliferation. Fibroblasts participate in angiogenesis through secretion of proteinases and deposition of ECM proteins. Reepithelialization starts and actively continues during the remodeling phase, characterized by collagen conversion and vascular regression. The release of TGF-β induces wound contraction and closure, and tissue remodeling, till the recovery of tissue integrity. When inflammation persists, wounds do not heal and chronic ulcers occur.

Wound healing involves a coordinated interaction of cells, proteins, growth factors, small molecules, proteases, and extracellular matrix (ECM) components aiming at restoring tissue morphology and functioning. The network of communications between stromal, endothelial, and immune cells is the key for determining the course of healing and recovery of tissue function and features (Rodrigues et al., 2019). Skin repair process can be divided into sequential phases: hemostasis, inflammation, proliferation, and remodeling (Figure 1). Although different growth factors, cytokines, and predominant cell types characterize each phase at different times, a considerable amount of overlap can occur (Sorg et al., 2017; Rodrigues et al., 2019). The four phases require different kinetics: 1) coagulation and hemostasis, starting immediately after injury; 2) inflammation, shortly after, lasting few days; 3) proliferation, occurring in several days; 4) wound remodeling, lasting days or even many months (Figure 1) (Reinke and Sorg, 2012; Velnar and Gradisnik, 2018).

The first response to a wound is constriction of the injured blood vessels, accompanied by activation of platelets and coagulation to form a fibrin clot to stop blood flow and provide a scaffold for incoming inflammatory cells. Indeed, in response to a mechanical injury, coagulation and hemostasis activate to prevent exsanguination and form a supportive matrix (including fibrin, fibronectin, vitronectin, and thrombospondins) that represents a substrate for invading cells, required later during the process. Upon injury, the microvasculature is disrupted leading to fluid accumulation, inflammation, and the development of hypoxia (Veith et al., 2019). At this stage, platelet-derived chemotactic factors released by platelet α-granules recruit leukocytes and monocytes to the area of injury to start the inflammatory phase finalized to tissue debris removal and bacteria killing (Etulain, 2018; Ridiandries et al., 2018). Leukocytes produce and release chemokines and cytokines (interleukin IL-1α, -1β, -6 and tumor necrosis factor-alfa, TNF-α), which, together with reactive oxygen species (ROS) release, amplify the inflammatory response (Reinke and Sorg, 2012; Veith et al., 2019). This second phase, often associated with edema, erythema, heat, and pain, aims to prepare the wound bed for the growth of new tissue. However, inflammation can become problematic if it is prolonged or excessive.

Once the lesion is cleaned out, the wound enters the third proliferative phase, where the aim is to fill and contract the wound edges and cover the gap. The proliferative phase is characterized by granulation tissue formation, collagen deposition, angiogenesis, and reepithelialization. Granulation tissue consists of layers of fibroblastic cells separated by a collagenous extracellular matrix containing capillary buds and inflammatory cells. The recruited neutrophils and macrophages release growth factors, such as transforming growth factor-beta (TGF-β), fibroblast growth factor-2 (FGF-2), platelet-derived growth factor (PDGF), and vascular endothelial growth factor (VEGF), responsible for activating resident ECs toward an angiogenic phenotype. Angiogenesis consists of EC proliferation, migration, and branching to form new blood vessels. Neovascularization, regulated predominantly by hypoxia-induced VEGF released by macrophages and fibroblasts, is needed to deliver nutrients and maintain the granulation tissue bed (Tonnesen et al., 2000, Li et al., 2003; Reinke and Sorg, 2012). Additionally, soluble factors induce endothelial precursor cells (EPC) recruitment in the granulation tissue and promote fibroblast proliferation and migration with changes in the ECM architecture. In this intermediate phase, macrophages continue to supply growth factors, promoting angiogenesis and stimulating resident fibroblasts to invade the wound and proliferate and remodel ECM through the synthesis of collagen, fibronectin, laminin, and metalloproteases, providing strength and elements to the injured tissue (Gurtner et al., 2008; Darby et al., 2014; Etulain, 2018; Ridiandries et al., 2018; Rodrigues et al., 2019). In the complex, the granulation tissue is composed by a high density of fibroblasts, granulocytes, macrophages, and capillaries (for the 60% of the mass) and loosely organized collagen bundles. It is well documented that angiogenic factors are present in wound fluid and promote repair, while antiangiogenic factors inhibit repair (Li et al., 2003; Schultz and Wysocki, 2009). During healing of the tissue defect, the edges of the wound are progressively brought together by the retraction of granulation tissue. This is due to the effect of TGF-β1 on fibroblasts which are induced to differentiate in myofibroblasts, contractile cells with stress fibers containing α-smooth muscle actin. This phenomenon, called wound contraction, is of great clinical importance in reducing the size of the wound. Reepithelialization simultaneously occurs and involves the proliferation of both unipotent epidermal stem cells from the basal layer and dedifferentiation of terminally differentiated epidermal cells (Gurtner et al., 2008; Rodrigues et al., 2019).

The final stage of the repair process, the remodeling or maturation phase, is characterized by the transition from granulation tissue to scar formation and maturation. Wound remodeling takes place when components of ECM undergo changes (e.g., replacement of collagen III by stronger collagen I) and myofibroblasts reduce the scar surface (Tomasek et al., 2002; Darby et al., 2014).

The process concludes with a decrease in cell density and a gradual arrest of angiogenesis, involving cell apoptosis and release of antiangiogenic factors (Reinke and Sorg, 2012; Sorg et al., 2018). When all these steps proceed in a regular and coordinated manner, a morphological and functional recovery of the injured tissue is obtained. However, if one or more of these mechanisms is impaired or delayed, healing is not guaranteed, and ulcer can occur with the risk of infections and chronic damage.

## Angiogenesis During Wound Healing

Among the different events that occur in the proliferative phase, angiogenesis is particularly important, because it forms the basis for tissue survival and recovery in the wound. Angiogenesis is defined as the formation of new vessels from preexisting ones and the microcirculation is the main site of vascular remodeling and angiogenesis. Angiogenesis appears to occur by two mechanisms, namely nonsprouting (intussusceptive) and sprouting angiogenesis (Ribatti and Crivellato, 2012). During intussusception, endothelial protrusions of opposing capillary walls extend towards each other and fuse creating an interendothelial contact (Burri et al., 2004). Sprouting angiogenesis is however the major form of neovascular growth, which requires migration, proliferation, and differentiation of ECs under the stimulation of specific glycoproteins called angiogenic factors, mainly VEGF and FGF (Hughes, 2008; Vandekeere et al., 2015). The development of blood vessels, which is a requirement for growth and regeneration, depends on a highly structured communication of ECs with their surrounding tissue. *In vivo* vascularization is based on complex cell-matrix and cell-cell interactions, where the ECM seems to play a pivotal role (Neve et al., 2014; Mongiat et al., 2016; Tracy et al., 2016).

The principal stimulus for angiogenesis occurrence is a lack of nutrients and oxygen, which characterize the hypoxic condition arising during tissue growth or following tissue injury and impaired blood flow (Ahluwalia and Tarnawski, 2012). Hypoxia is indeed able to promote hypoxia inducible factor1-α (HIF1-α) upregulation at nuclear level, which is responsible for angiogenic factor overexpression and vasoactive molecules upregulation. Under the action of the angiogenic factors VEGF, FGF, and PDGF, ECs undergo receptor activation and modification of intracellular pathways and cytoskeleton structure, leading to cell proliferation and chemotaxis (Ahluwalia and Tarnawski, 2012).

The first step is the binding of proangiogenic factors including VEGF, FGF-1 and 2, PDGF, and angiopoietins and stromal-derived growth factor (SDF-1), to their receptors on ECs of existing vessels, triggering complex and intricate intracellular signaling cascades. Heparin sulfate proteoglycans and syndecans also play a key role in regulating the angiogenic activity of VEGF and FGF-2 (Corti et al., 2019). Activated ECs secrete matrix metalloproteinases (MMPs) to degrade the capillary basement membrane (BM) and allow their migration and proliferation outside of the original blood vessel. Therefore, new vessels are formed as capillary sprouts and are then extended and remodeled. Finally, ECs interconnect to form a loop or a tube and the recruitment of pericytes stabilizes the newly formed vessels in a mature conformation (Carmeliet, 2003; Sorg et al., 2018).

In this framework, EC migration plays an important role for vascular remodeling and is a necessary condition for angiogenesis to occur. EC migration is a coordinated process that involves changes in cell adhesion, signal transduction, and cytoskeleton dynamic reorganization (Li et al., 2003; Velnar and Gradisnik, 2018).

The regulation of this process is achieved by three types of mechanisms: chemotaxis or migration towards a concentration gradient of soluble chemoattractants; haptotaxis or rather migration in response to a gradient of immobilized ligands; and mechanotaxis which is the migration induced by mechanical forces. Specifically, chemotaxis of ECs is driven by growth factors (VEGF and FGF-2, among the most important), haptotaxis is related to increased EC migration activated in response to integrins (e.g., αvβ3 and αvβ5), bound to ECM components, and mechanotaxis is associated to a polarization of cytoskeleton and cell-ECM interactions in the blood flow direction (Lamalice et al., 2007).

Due to the combined action of cell migration and proliferation, the nascent vessels grow in the hypoxic/ischemic wounded tissue particularly rich of angiogenic stimuli. Circulating EPCs concur to the nascent vessels, being recruited by the factors released by the hypoxic environment, primarily VEGF and SDF-1 (Zhu et al., 2016). The process arrives at its end when the bud cavitates and blood starts to flow, bringing oxygen and nutrients to the tissue and taking away CO_2_ and waste catabolites.

### The Complexity of Wound Angiogenesis

Sprouting angiogenesis has recently been a subject of intense research since it is a requirement for growth and regeneration. The process has many sequential hierarchical steps that require the close interaction of EC with both cellular and acellular components of the surrounding tissue (Hughes, 2008). *In vivo*, capillaries are embedded in a microenvironment that consists of the ECM and cellular components including fibroblasts as well as immune cells. The ECM is a complex, noncellular network constituted by distinct components that is found in two different locations, i.e., the interstitium as interstitial ECM and, in association with epithelial and endothelial tissues, as the BM (Neve et al., 2014).

During early phases of wound healing, capillary sprouts invade the fibrin/fibronectin-rich wound clot and within a few days organize into a microvascular network throughout the granulation tissue. Growth factors that are released from the ECM trigger sprouting angiogenesis. These include a range of angiogenic factors, the most important being VEGF-A (Henning, 2016). Angiogenic stimuli activate the ECs to migrate into the avascular tissue (Eming and Hubbell, 2011). ECs express VEGF receptor-2 (VEGFR-2) that responds to the VEGF-A gradient. Once an angiogenic stimulus occurs, MMPs break down the BM of the blood vessel, mainly near the trigger sites (Davis and Senger, 2008). During sprouting, ECs are triggered by the VEGFR-2/VEGF-A binding to temporarily transform into migrating tip cells. These cells are polarized and have well-developed filopodia that enable them to interact with the ECM via integrins. These proteins (primarily αvβ3) of the ECs filopodia surface have an adhesive function during the endothelial migration (Davis and Senger, 2008). ECM proteins are important for adhesion and migratory processes of the endothelial tip cells and therefore promote angiogenesis (Neve et al., 2014; Mongiat et al., 2016). The endothelial tip cells move into the surrounding avascular extracellular matrix towards the angiogenic stimulus (Stratman et al., 2009). To enable this process, the MMPs form tunnels in the ECM to facilitate endothelial migration (Sacharidou et al., 2012).

Membrane-type 1-matrix metalloproteinases (MT1-MMP), synthesized by the ECs themselves, are responsible for most of the proteolysis of the ECM (Stratman et al., 2009). Endothelial stalk cells follow the tip cells into the ECM where they proliferate and elongate the developing capillary sprout as well as establish its internal lumen. The tubular lumen is formed by intraendothelial vacuoles that fuse. For the development of the vacuoles, MT1-MMP and the integrins αvβ3 and α5β1 play important roles (Welch-Reardon et al., 2014). Tight and adherens cell junctions are established between the stalk cells of the newly built tube and, consequently, a new vessel arises.

Due to their roles in cell-matrix interactions and especially matrix remodeling, fibroblasts are crucial in vascular development through transmitting biochemical signals and mechanical forces that affect cell survival, shape, and orientation (Kamei et al., 2006; Costa-Almeida et al., 2015). Stalk ECs synthesize, in cooperation with surrounding fibroblasts, basement membrane proteins, namely laminin, collagen IV, perlecan, nidogen, collagen XVIII, and fibronectin. The BM envelops and stabilizes the newly developing capillary sprout, serving as an acellular barrier against the capillary microenvironment and ensuring the correct polarity of ECs (Davis and Senger, 2008). Maturation and stabilization as well as remodeling of the dynamic capillary structures follow initial angiogenesis. As tubules mature, their ECs transform into quiescent phalanx cells (Senger and Davis, 2011). When collagen accumulates in the granulation tissue to produce scar, the density of blood vessels diminishes.

In dermal wounds with robust healing, the angiogenic activity during the proliferative phase initially creates a disorganized vascular network with highly tortuous vessels pathways, often reaching higher vessel numbers than normal (DiPietro, 2016). Following this peak in neovascularization, there is increased expression of antiangiogenic factors, such as Sprouty2 and pigment epithelium-derived factor (PEDF), leading to vascular regression and pruning (Wietecha et al., 2011; Wietecha et al., 2015). Maturation and stabilization of the new vascular network require the involvement of pericytes and vascular smooth muscle cells (Bergers and Song, 2005). A key growth factor is PDGF-BB, which acts on pericyte differentiation (Hellberg et al., 2010). Additionally, there is the contribution of angiopoietin-2/Tie2 receptor in pericytes which regulate angiogenesis and maturity of vascular networks (Teichert et al., 2017).

### Signaling Pathways in Endothelial Cells During Angiogenesis

ECs receive multiple stimuli from the environment that eventually induce them to progress along all the stages of angiogenesis, until new vessels are formed in the wound bed. Angiogenic signals that promote EC survival, proliferation, migration, and finally differentiation are the result of a complex framework, involving cell-ECM and cell-cell interactions, and action of soluble mediators (Muñoz-Chápuli et al., 2004; Lamalice et al., 2007). ECs express a large number of receptors that make them responsive to several growth factors and cytokines involved in the promotion of angiogenesis, but the most important and specific for their action is VEGFR (Hofer and Schweighofer, 2007). In particular, the binding of VEGF-A, secreted by cells in the hypoxic environment, to VEGFR-2 is the major way by which EC migration is promoted (Olsson et al., 2006). Specifically, like other tyrosine kinase receptors, VEGFR-2 undergoes ligand (VEGF-A) induced dimerization and oligomerization, which activates its intrinsic tyrosine kinase activity, resulting in auto- and transphosphorylation on specific tyrosine residues in the cytoplasmic domain, ultimately being responsible for cell proliferation and migration. These pathways involve activation of the small GTPases of the Rho family, PI-3K/Akt, p38 MAPK, FAK, and ERK1/2 signaling cascades (Muñoz-Chápuli et al., 2004; Webb et al., 2004; Lamalice et al., 2007; Yang et al., 2017).

Intermediate messengers are upregulated during angiogenic cell activation as gasotransmitters nitric oxide (NO) and hydrogen sulfide (H_2_S). PI-3K/Akt-activated eNOS produces NO, which is a major regulator of EC migration and angiogenesis, by inducing expansion of EC surface, after vasodilatation, associated with a more proper response of endothelium to angiogenic and promigratory stimuli (Dimmeler et al., 2000; Morbidelli, 2016b). In addition, a particular attention has been put on hydrogen sulfide (H_2_S). Endothelial-associated H_2_S producing enzymes are activated or upregulated by hypoxia and VEGFR-2 signaling cascades, as recently reviewed (Ciccone et al., 2021).

### Endothelial Dysfunction and Impaired Healing

All the above mechanisms however are working in healthy endothelial cells. Physiological conditions such as ageing and pathologic conditions as metabolic syndrome or diabetes are characterized by endothelial impaired functions, called endothelial dysfunction, with reduced ability of ECs to release vasoactive and protective factors like NO and to undergo beneficial and sufficient angiogenesis. These disorders are indeed accompanied by altered healing, chronic ulcers, and infections that in extremis may need amputation (Brem and Tomic-Canic, 2007). Beside the inability of ECs to promote a reparative angiogenesis, we have to consider the microenvironment composition. Recent analysis of the exudate of venous nonhealing leg ulcers has found increased antiangiogenic factors, increased VEGF proteolytic products, and increased levels of soluble VEGFR-1, known to neutralize VEGF-A activity (Lauer et al., 2000; Drinkwater et al., 2002; Eming et al., 2004; Eming et al., 2010).

All these findings push scientists to identify novel biochemical targets for the development of therapies to promote endothelial proper functions during reparative angiogenesis. The research is nowadays active in characterizing novel drugs or therapies and their delivery systems to be used in pathological conditions characterized by endothelial dysfunction and impaired angiogenesis and wound healing.

## Physical-Chemical Factors Controlling Wound Healing

The different steps of tissue repair are strictly regulated by a multitude of biochemical and physical factors, including gravitational/mechanical forces acting at cellular and tissue level. Interruption, failure, or alteration in one or more phases of the repair process can lead to the formation of nonhealing chronic wounds or fibrotic scars, accompanied by pain and inflammation (Guo and DiPietro, 2010).

In general terms, the factors that influence repair can be categorized into local and systemic. Local factors are those directly influencing the characteristics of the wound itself like oxygenation/hypoxia, infection occurrence, vascular insufficiency, or presence of foreign bodies. On the other hand, systemic factors are the overall health or disease state of the individual affecting his or her ability to heal, such as age and gender, stress, nutrition, alcoholism and smoking, immunocompromised conditions, and diseases (as diabetes) (Guo and DiPietro, 2010). Although many factors related to a patient’s conditions cannot be changed, local factors can be controlled and improved in order to obtain the best therapeutic result.

Among the exogenous factors, there are mechanical stressors as pressure, vibrations, and loading. ECs are particularly sensitive to changes in gravitational forces and the mechanisms of mechanotransduction are now known (Maier et al., 2015; Li et al., 2018).

In particular, studies on unloading condition can be performed at different levels as single cell, cell cocultures or organoids, and living animals, as rodents or invertebrates (leeches), exposed to real microgravity in vehicles operating in terrestrial orbits or to terrestrial models of unloading (Cialdai et al., 2020). Animal hindlimb unloading (HLU) or bed rest experimental studies in humans are established models designed to mimic unloading conditions in space.

### *In Vivo* Experiments of Unloading Effect on Wound Healing

Very few are the reports on the effects of mechanical unloading and physical deconditioning on wound healing *in vivo*. Several studies have indicated that spaceflight can adversely affect tissue repair in muscle and bone (Pospishilova et al., 1989; Ilyina-Kakueva and Burkovskaya, 1991; Kaplansky et al., 1991; Bolton et al., 1997; see Genah et al., 2021a for review). In relation to skin repair, Davidson et al. (1998) conducted a study to determine the effects of microgravity on wound healing in rats, in term of granulation tissue and collagen formation. They found a reduced capacity of wounds to heal, being the cells less responsive to added growth factors.

Radek et al. demonstrated that in rats exposed for 2 weeks to HLU before excisional wounding, the healing process was delayed on day 2 with respect to ambulatory controls. Although the levels of proangiogenic growth factors FGF-2 and VEGF were similar between the two groups, wound vascularization in HLU animals was significantly reduced at day 7. To further examine this disparity, total collagen content was assessed and found to be similar between the two groups (Radek et al., 2008). Recently, inhibition of cell proliferation and angiogenesis was verified in skeletal muscles of rats exposed to hindlimb unloading through RNA sequences analysis (Cui et al., 2020).

Taken together, these results suggest that keratinocyte and EC functions may be impaired during the wound healing process under periods of prolonged inactivity or bed rest.

### Effect of Weightlessness on Endothelial Cell Transcriptome, Morphology, and Function

**FIGURE 2:**
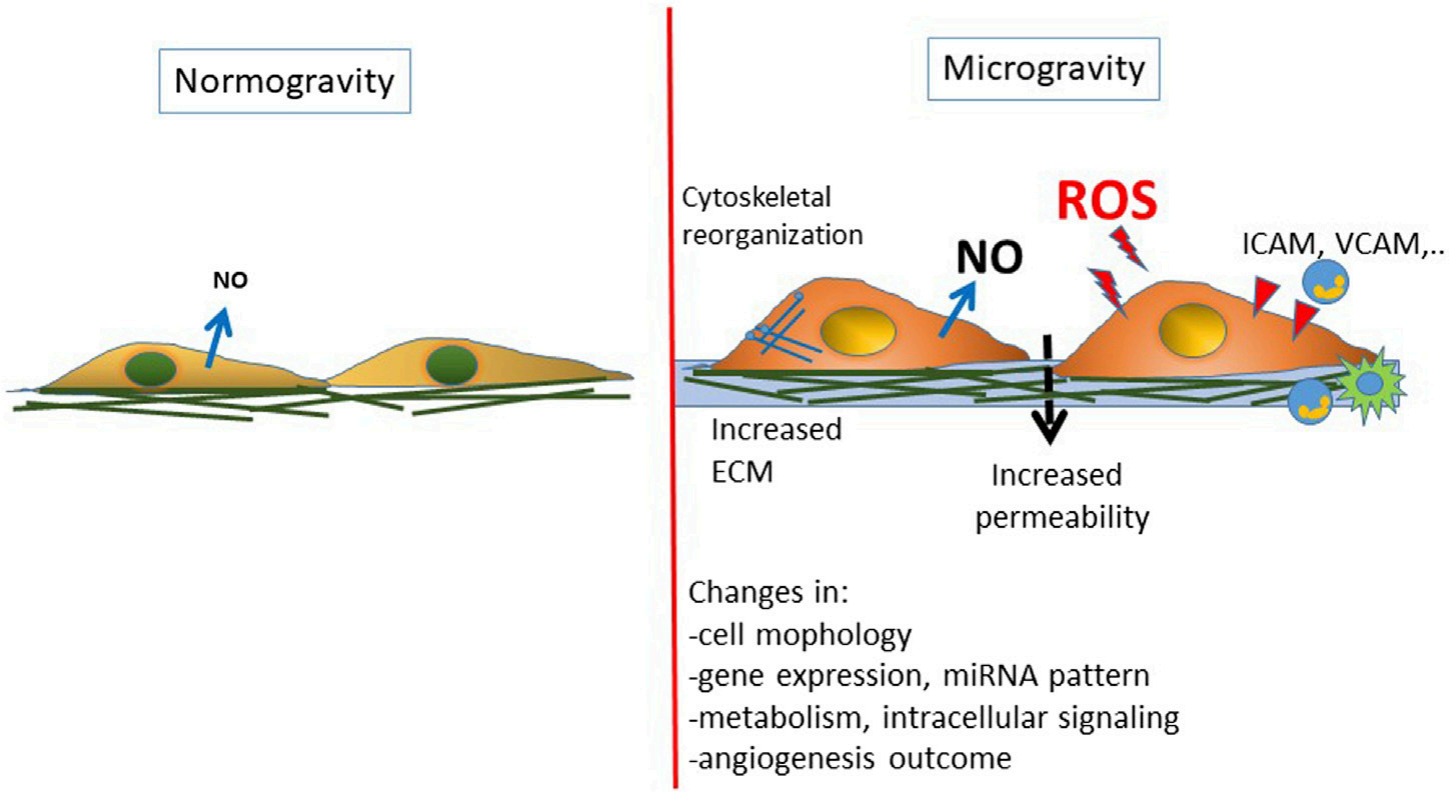
Schematic representation of the changes reported on endothelial cells exposed to real or simulated microgravity. A general condition of endothelial dysfunction is found associated with an inflammatory/prooxidative phenotype.

ECs are mechanosensitive cells undergoing morphological and functional changes in response to fluid shear stress, cyclic tensile strain, and substrate stiffness. In space their alterations contribute to cardiovascular deconditioning and immune dysfunction commonly faced by astronauts during spaceflight. Ground-based and space experimentation has provided a body of evidence about how ECs can respond to the effect of simulated and real microgravity (Byfield et al., 2009; Chancellor et al., 2010; Maier et al., 2015; Li et al., 2018). Exposure of ECs to microgravity in space can change morphology and gene expression, displaying heterogeneous cell size and shape (Kapitonova et al., 2012), 3D growth (Pietsch et al., 2017), energy and protein metabolism deficiency (Chakraborty et al., 2018), significant suppression of genes associated with host defense (Chakraborty et al., 2014), and alterations in genes involved in cell adhesion, oxidative phosphorylation, and stress responses (Versari et al., 2013) (Figure 2). To date, only a limited number of studies in space have been performed and the impact of real microgravity on EC functions still remains unclear.

Most of the data derive from ground-based microgravity simulators, as rotating wall vessel (RWV) (a 2D clinostat) (Goodwin et al., 1993) and random positioning machine (RPM) (a 3D clinostat) (Morbidelli et al., 2005; Wuest et al., 2017).

Specifically, markers for leukocyte adhesion and recruitment, adhesive counterreceptors and inflammatory cytokine expression pattern are altered in RWV (Wang et al., 2015), documenting a proinflammatory phenotype. Nevertheless, the outcomes are controversial in literature. For instance, a decreased expression of intercellular adhesion molecule-1 (ICAM-1) has been found in ECs cultured in RPM for 24 h (Grenon et al., 2013). However, upregulation of ICAM-1 transcription was found after 30 min clinorotation and the clustering of ICAM-1s on cell membrane was observed when ECs have been activated by TNF-α and cultured in RWV (Zhang et al., 2010).

Mechanistically, the cytoskeletal remodeling has been considered the transducer of cellular responses to microgravity (van Loon, 2009; Long et al., 2015). The reorganization of cytoskeleton proteins following microgravity exposure includes microtubule and actin filament (F-actin) upregulation (Zhang et al., 2017; Buravkova et al., 2018). Significantly downregulated amount of actin (Carlsson et al., 2003; Corydon et al., 2016), depolymerization of F-actin (Kang et al., 2011), and clustering of the stress fibers at the cellular membrane and around the nucleus (Infanger et al., 2007; Grenon et al., 2013) have been reported, and the actin rearrangement is typically RhoA dependent (Shi et al., 2017). The controversial results probably depend on the EC type used in the experiments, stimulation facility, and time of exposure.

Cytoskeletal rearrangement was accompanied by the overexpression of ECM proteins, including collagen I, fibronectin, osteopontin, and laminin (Infanger et al., 2006; Grimm et al., 2009; Buravkova et al., 2018), but again also downregulation has been described (Corydon et al., 2016).

Furthermore, intracellular signaling and cell–cell communication are crucial for EC functional alterations in microgravity. For example, NO has been reported to be upregulated by RPM and is deemed to be responsible for angiogenesis and cardiovascular deconditioning experienced by astronauts during spaceflight (Siamwala et al., 2010; Grenon et al., 2013). The increased eNOS activation found in 2D or 3D clinostat cultures in ECs seems to be due to PI-3K pathway (Shi et al., 2012), actin remodeling (Siamwala et al., 2010), and caveolin-1- (Cav-1-) mediated mechanotransduction (Shi et al., 2016).

Concerning angiogenesis outcome, in agreement with the results reported by other authors (Kang et al., 2011; Xu et al., 2018), our studies demonstrated that microgravity induces significant changes in EC behavior with reduced cell survival, induction of apoptosis, and angiogenesis impairment (Morbidelli et al., 2005; Maier et al., 2015).

Controversial results were however reported both in differentiated ECs depending on the district (micro- or macrocirculation) and in mesenchymal stem cells (MSC). Simulated microgravity promotes angiogenic output in HUVEC via RhoA-dependent rearrangement of actin and cytoskeleton (Shi et al., 2017). It has been demonstrated that microgravity could stimulate mature ECs and MSC to produce IL-8 and VEGF as well as other paracrine factors involved in angiogenesis. This is responsible for the regulation of EC functions by creating a specific microenvironment in support of EC proliferation and migration (Dittrich et al., 2018; Ratushnyy et al., 2018).

Recently, in human ECs cultured under simulated microgravity achieved with a clinostat, a total of 1,870 miRNAs were found to be differentially expressed with respect to normal gravity. The functional association of identified miRNAs targeting specific mRNAs revealed that a series of miRNAs (hsa-mir-496, hsa-mir-151a, hsa-miR-296-3p, hsa-mir-148a, hsa-miR-365b-5p, hsa-miR-3687, hsa-mir-454, hsa-miR-155-5p, and hsa-miR-145-5p) differentially regulated the genes involved in cell adhesion, angiogenesis, cell cycle, JAK-STAT signaling, MAPK signaling, NO signaling, and VEGF signaling, finally favoring angiogenesis (Kasiviswanathan et al., 2020). In a study on endothelial progenitor cells, cultured in simulated microgravity, a facilitation of functional angiogenic properties has been reported, with increased HIF-1α and eNOS/NO induced FAK/ERK1/2 pathway upregulation in HUVEC (Kong et al., 2021).

In real gravity study, cultured ECs were kept on board of the SJ-10 Recoverable Scientific Satellite for 3 and 10 days (Li et al., 2018). Space microgravity suppressed the glucose metabolism; modulated the expression of cellular adhesive molecules such as ICAM-1, vascular cell adhesion molecule-1 (VCAM-1), and CD44; and depressed the secretion of proangiogenic factors and proinflammatory cytokines. Moreover, space microgravity induced the depolymerization of actin filaments and microtubules, promoted vimentin accumulation, restrained collagen I and fibronectin deposition, regulated the mechanotransduction through focal adhesion kinase and Rho GTPases, and enhanced the exosome-mediated mRNA transfer. As previously seen in simulated microgravity, neither three-dimensional growth nor enhanced NO production has been observed in real microgravity (Li et al., 2018).

Moreover, some preliminary results from *in vitro* studies in modeled microgravity indicate that the cross-talk between fibroblasts and ECs, a building block in the healing evolution, is impaired. The production of angiogenic growth factors is altered as well (Cialdai et al., 2017), with a consequent inability of ECs to form tube-like structures. The complex cross-talk between fibroblasts and ECs and its role in tissue healing are beyond the focus of the present paper.

Despite these controversial results in cultured cells, probably due to different cell source and microgravity protocols, astronauts spending a long time in International Space Station (ISS) are more vulnerable to vasculopathies, associated to endothelial dysfunction (Zhang and Hargens, 2018; Garrett-Bakelman et al., 2019; Navasiolava et al., 2020), thus strengthening the finding of defective angiogenesis and tissue repair in space environment.

## Pharmacological, Cellular, and Physical Countermeasures

In current clinical practices, a series of drugs can be employed to control symptoms related to wound healing like inflammation, oedema, pain, and steroidal or nonsteroidal anti-inflammatory drugs. Furthermore, dressings and topic products are used to create and keep a humid environment, providing the ideal conditions for a correct wound healing process (Dreifke et al., 2015). However, side effects, or even opposite effects on wound healing, such as hypertrophic scarring, contraction, and necrosis, can limit their employment, especially considering their long-term use, raising the necessity for alternative countermeasures (Dreifke et al., 2015).

Emerging skin regeneration techniques involving scaffolds activated with growth factors, bioactive molecules, and genetically modified cells are being exploited to overcome wound healing technology limitations and to implement personalized therapy design. Results are however partial and under consolidation. The following sections report the state of the art or the ultimate findings on various types of countermeasures for recovery of tissue integrity (Table 1). However, not all these countermeasures can be adopted in a space environment, due to their shelf life and stability in space, the necessity to be produced/manipulated in real time by highly prepared operators or in dedicated facilities, and the consideration of the specific personal need.

**TABLE 1:** Countermeasures to improve or accelerate wound healing through enhanced angiogenesis.

Type of therapies	Specific clues
Protein therapeutics	Growth factors: PDGF, EGF, FGF, and VEGF
	Nongrowth factor proteins/glycoproteins: insulin, erythropoietin, SDF-1, syndecans
	Peptides: antimicrobial peptides, vasointestinal peptide
	Blood-derived factors: PRP, hemoglobin
Gene and nucleic acid-based therapies	Gene therapy for growth factors microRNA
Drugs and bioactives	Statins
	NO donors
	ACE inhibitors
	Natural compounds (astragaloside, centelloids, and asiaticoside)
	Nutraceuticals
Polymers for dressing or scaffold preparations	Dextran hydrogels
	Hyaluronan oligosaccharides
Stem cell–based therapies (in the form of naïve or genetically modified cells, cell secretome, exosomes, and EV)	Adipose-derived stem cells
	Bone marrow–derived mesenchymal stem cells
	Induced pluripotent stem cells
	Endothelial precursor cells
Physical therapies	Ultrasound/low pressure shock-waves
	Laser therapy/photobiomodulation
	Electrical stimulation
	Hyperbaric oxygen
	Vacuum-assisted closure
	Hypergravity

For details on the state of the art of the single therapies, see Dreifke et al. (2015) and Veith et al. (2019). For hypergravity use in unloading conditions, see *Physical Countermeasures* section.

### Pharmacological Countermeasures

As reported above, different growth factors as FGF, VEGF, EGF, and PDGF are needed to orchestrate neovascularization and the whole wound healing process and many attempts have been made to use recombinant growth factors in dermal wound healing with different approaches and formulations (see Veith et al., 2019 for a comprehensive review). To the best of our knowledge, there are only a few drugs of protein nature approved to promote wound healing with potential proangiogenic properties. The first is represented by recombinant PDGF prepared as a skin preparation (Regranex Gel, 0.01% becaplermin). Regranex Gel is the first and only FDA-approved recombinant PDGF therapy for use on diabetic neuropathic ulcers. Regranex contains becaplermin, a human PDGF that is indicated for the treatment of lower extremity ulcers that extend into the subcutaneous tissue or beyond and have an inadequate blood supply. PDGF initiates healing by attracting repair cells to revitalize wounds (Pierce et al., 1991). Indeed, PDGF works by stimulating fibroblast proliferation, increasing granulation tissue and the rate of reepithelialization and revascularization, and promoting collagen production (Heldin and Westermark, 1999).

Other attempts have included studies with gene therapy or recombinant growth factors administered directly in wounds or delivered with scaffolds, nanomaterials or cells (Veith et al., 2019), but the results still need to be confirmed both in clinical studies on an appropriate number of patients/pathological conditions and, on top of this, in a space environment.

**FIGURE 3:**
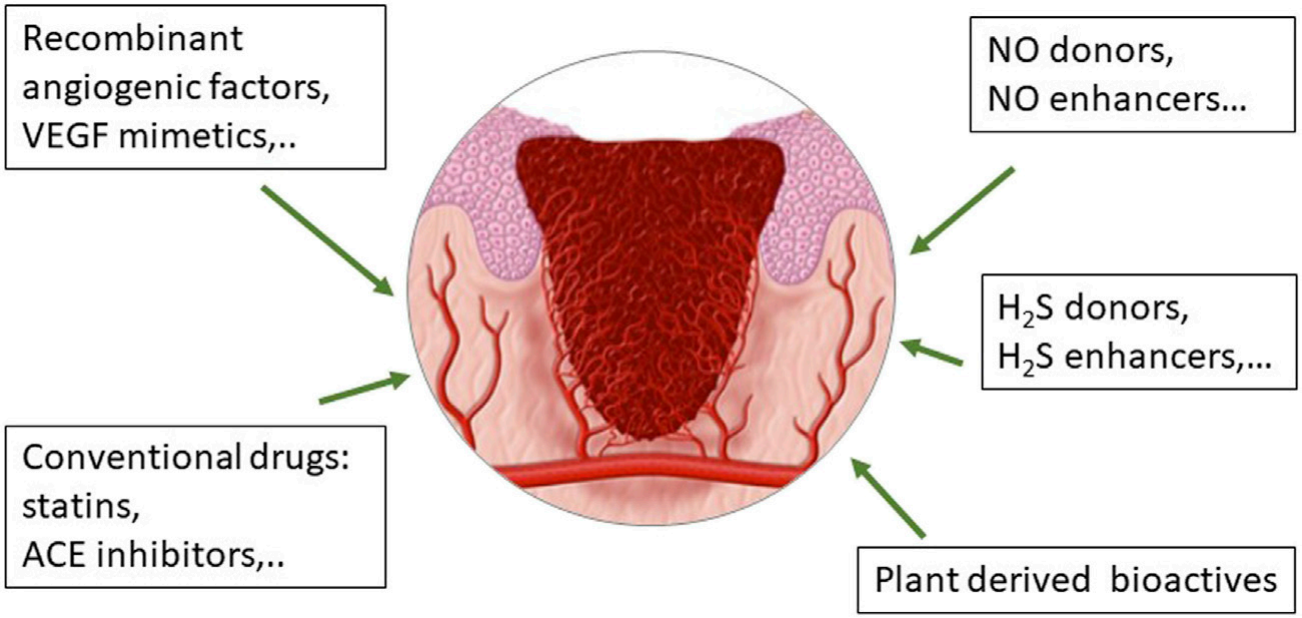
Drugs in use or under development to improve wound healing through their effect on endothelial functioning and angiogenesis, crucial events during the formation, and evolution of the granulation tissue.

Conventional drugs are small molecules that could be an optimal alternative to recombinant growth factors to be used in space, since many of them are part of the on-board pharmacy and their pharmacological and toxicological characterization is robust (Figure 3). Among the small molecules, particular attention has been posed to statins, widely used to lower cholesterol level due to their inhibitory activity on the liver enzyme 3-hydroy-3-methyl-glutaryl-coenzyme A reductase. Statins are molecules with pleiotropic and multitarget activity, demonstrating a high protective profile on ECs and anti-inflammatory and antioxidative properties, among others (Khaidakov et al., 2009) (Figure 3). Statins have been established to possess proangiogenic features, protecting the cardiovascular system against ischemic injury (Kureishi et al., 2000). Simvastatin has been characterized for its healing activity in experimental diabetic ulcers, demonstrating a proangiogenic action with NO pathway upregulation (Bitto et al., 2008). The efficacy of statins has been demonstrated through daily oral administration and by topical treatment, being able not only to produce neovascularization but also lymphangiogenesis (Asai et al., 2012). Recently, novel drug delivery systems for statin local release are under evaluation, demonstrating their efficacy and potential clinical application in chronic ulcers (Rezvanian et al., 2016; Yasasvini et al., 2017). The research on the best formulation and treatment protocols is still active and no report has explored their employment in space environment.

As stated above, NO has been identified to exert a pivotal role in wound healing. NO levels increase significantly following skin injury and then gradually decrease as healing progresses (Childress and Stechmiller, 2002). At the same time pathological conditions characterized by H_2_S deficiency as diabetes are known to be associated with impairment of skin healing and progressive ulcerations (Ciccone et al., 2021). These considerations encouraged us to develop strategies with the ability to release both gasotransmitters in a tunable manner for the control of endothelial function and angiogenesis.

Considering the central role of NO in angiogenesis, we have contributed to demonstrate the beneficial effect on endothelial cell functions and angiogenesis by a series of synthetic molecules: 1) a peptidomimetic of VEGF, as QK; 2) the NO donors metal NONOates; and 3) the angiotensin converting enzyme (ACE) inhibitor zofenoprilat, which shares the common feature of releasing NO and H_2_S in a controlled manner (Finetti et al., 2012; Monti et al., 2016; Monti et al., 2018) (Figure 3).

The peptidomimetic analogue of VEGF, QK, reproduced all the angiogenic functions of the whole molecule, including its ability to promote the eNOS pathway (Finetti et al., 2012). Being a peptidomimetic, QK could be a promising tool to be used in space, for its easy manipulation and stability.

The metal-nonoate Zn(PipNONO)Cl shows an interesting kinetic of NO release (both fast and prolonged) due to the ability to upregulate eNOS and H_2_S producing enzymes in ECs, thus contributing to improve endothelial function (Monti et al., 2018). Its role in human skin tissue repair is under evaluation within space agencies programs (LM personal communication). The H_2_S facilitating properties on tissue repair have been recently strengthened by the findings that H_2_S improves wound healing by induction of angiogenesis (Ciccone et al., 2021) and restoration of EPC functions in type 2 diabetic mice (Liu et al., 2014).

Concerning conventional drugs used in clinics for other purposes, we have demonstrated endothelium protective, proangiogenic, and anti-inflammatory properties by the active moiety of the ACE inhibitor zofenopril, namely zofenoprilat, which through its SH group behaves as a H_2_S donor and promoter (Monti et al., 2013; Monti et al., 2016) (Figure 3). Its indication in wound healing however has never been verified.

Beside the recombinant growth factors and conventional drugs, an interesting therapeutic approach for wound healing management is represented by ethnopharmacology and traditional remedies, which are present in the various cultures and provide preparations and active principles with anti-inflammatory, antibacterial and proproliferative/proangiogenic properties (Morbidelli, 2016a; Morbidelli et al., 2018) (Figure 1). Plant-derived active principles have been isolated and demonstrated to reduce scar formation as astragaloside IV from *Astragalus membranaceus* and centelloids and asiaticoside from *Centella asiatica* (Chen et al., 2012; Bylka et al., 2014). Concerning the beneficial effects of Mediterranean diet, olive oil–based diet has been demonstrated to improve cutaneous wound healing of pressure injury in mice through the reduction of inflammation and stimulation of redox equilibrium (Schanuel et al., 2019). In this scenario, we have contributed to evaluate the protective effect of extra-virgin olive oil polyphenol hydroxy-tyrosol and its metabolic derivative which positively controls angiogenesis and the endothelial-to-mesenchymal transition (Terzuoli et al., 2020). Another example of nutraceuticals which exerts antioxidant and protective effects on EC function affected by high glucose is erucin, derived from *Eruca sativa* Mill. seeds. Its endothelial positive outcomes are correlated to increased H_2_S intracellular levels (Martelli et al., 2021). Studies are needed to further validate these findings in experimental models and in the clinic and to define other nutraceuticals with protective effects on ECs and promoting reparative angiogenesis.

### Cell Therapies

The use of stem cells, alone or by the help of a scaffold, provides better and faster healing of burn wounds, with decreased inflammation, slow scar progression, and reduced fibrosis. Stem cell homing at the wound site results in granulation tissue formation, immunomodulation, neovascularization, apoptosis inhibition, and induction of epithelial cell proliferation with skin regeneration. While these findings are clear in animal models, the validation of their clinical use is at the beginning (Veith et al., 2019; Phua et al., 2021). Sources of MSCs in adults are bone marrow, adipose tissue, and umbilical cord blood. Additionally, induced-pluripotent-stem-cell- (iPSC-) derived MSC have been reported to be suitable for cell therapy. However, their effectiveness in human patients remains to be established (Jo et al., 2021; Mazini et al., 2021; Ullah et al., 2021). The proposal of proangiogenic paracrine secretion by stem and precursor cells cultured in microgravity has been provided (Ratushnyy et al., 2018; Kong et al., 2021).

Dermal fibroblasts are the major cell type in skin dermal layers. As said above, they actively participate in skin regeneration and these cells are becoming attractive candidates for cell-based therapies in wound healing. Due to their heterogeneity linked to variable activation by inflammatory stimuli, tissue niche of origin and different scar forming properties, their potential clinical exploitation is far (Xue et al., 2021).

Due to the plethora of secretory products involved in angiogenesis (as VEGF and PDGF), tissue remodeling, and wound healing (Pierce et al., 1991; Frechette et al., 2005; Lacci and Dardik, 2010), platelet therapy has been applied in regenerative medicine and wound healing from decades (Martinez-Zapata et al., 2016). Recently, in cultured fibroblasts and in an experimental model of dermal injury in leech exposed to clinostat induced microgravity, we have demonstrated the efficacy of platelet rich plasma (PRP) in accelerating healing by acting on fibroblast migration (Cialdai et al., 2017; Cialdai et al., 2020).

While the use of cells in a space environment seems not feasible due to the many risky procedures (self-harvesting, *in vitro* culture and checking of safety, inoculation in the wound or systemically), the use of autologous PRP appears a very promising approach in the astronauts not only for dermal repair but also for regenerative bone, tendon, and endodontic treatment, where improvement of angiogenesis is a necessary step for proper repair.

### Microvesicles and Exosomes

Recently, a therapeutic role for extracellular vesicles (EV) derived from stem cells has been described in animal wound models (Dalirfardouei et al., 2021). Extracellular vesicles and in particular exosomes contain various bioactive molecules as proteins, enzymes, and nucleic acids, thus providing the wound with all the necessary stimuli to promote angiogenesis and cell proliferation and regulate inflammation and collagen remodeling, ultimately leading to tissue healing (Table 1). The use of stem-cell-derived exosomes seems more feasible with respect to cell therapy, without potential problems related to the use of living cells, which would make their use in space quite impossible. Additionally, they are not rejected by the immune system and have a homing effect and their dosage can be easily controlled. *In vivo* studies on animal models demonstrate the beneficial effects of EVs on accelerating wound closure and reepithelization in a dose-dependent manner. Various studies demonstrate induction of angiogenesis through the conventional mechanisms of PI-3K/Akt, MAPK/ERK1/2, and JAK/STAT pathways. Interestingly, the upregulation of TGF-β2/SMAD2 pathway involved in scar inhibition has been reported (Dalirfardouei et al., 2021). They can also act as carriers for other interventions and be combined with scaffolds. Also this innovative approach should be, however, thoroughly verified before clinical utilization in particular with respect to EV manufacturing, treatment protocols, and long-term follow-up (An et al., 2021).

### Physical Countermeasures

In recent years, many therapies aiming at stimulating the healing process are in clinical use, such as ultrasound, laser therapy, and other forms of photobiomodulation, electrical stimulation, hyperbaric oxygen, and vacuum-assisted closure (Dreifke et al., 2015; Nesi-Reis et al., 2018; Priyadarshini et al., 2018; Micheli et al., 2019; Xu et al., 2021). The efficacy of physical approaches is due to the fact that all the cell types involved in wound healing (fibroblasts, keratinocytes, and ECs) are sensitive to mechanical forces at cellular and molecular level acting as mechanotransducers.

ECs in particular respond to mechanical stimuli such as shear, strain, and stretch. This property can be exploited to induce reparative angiogenesis in pathological conditions associated with insufficient angiogenesis. In cultured ECs and in a mouse model of wound healing, it has been demonstrated that low-pressure shock waves induced angiogenesis. Increased EC migration and proliferation were associated with enhanced Ca^++^ influx and PI-3K which is usually observed when ECs are exposed to stretch. Shock wave treated mice showed enhanced wound-induced angiogenesis documented by increased vascular area and vessel length. Accelerated wound closure was observed compared to control mice (Sundaram et al., 2018).

Photobiomodulation, i.e., lasers emitting red/IR radiation, promotes angiogenesis in wound healing (de Madeiros et al., 2017). In a previous experimental paper, ECs were exposed to simulated microgravity or pulsed Nd:YAG laser radiation to assess their behaviour and morphology. Increased fibronectin and laminin could be the cause for impaired ECM rebuilding and altered cell adhesion/migration in microgravity (Monici et al., 2011) in accordance with previous data on impairment of angiogenesis (Morbidelli et al., 2005). On the contrary, the exposure to Nd:YAG laser pulses induced the formation of a highly ordered array of fibronectin fibrils on EC surface and cell spreading to form a monolayer (Monici et al., 2011). Recently, we have additionally demonstrated the anti-inflammatory effect of NIR laser on dermal fibroblasts stimulated with inflammatory cytokines, through an inhibition of NF-kB transcription pathway (Genah et al., 2021b). All these results suggest a beneficial effect of photobiomodulation as an effective healing option with proangiogenic and anti-inflammatory properties. Its inflight applicability should however be verified.

Among different countermeasures implemented to minimize the effects of microgravity, a promising one could be artificial gravity. We have demonstrated that discontinuous hypergravitational stress did not significantly affect cell survival in macrovascular and microvascular ECs. In both cell populations, we found similar changes in cytoskeleton and αvβ3 integrin distribution that in microvascular ECs were combined with an increased anaerobic metabolism and cell detachment from the substratum (Monici et al., 2006; Morbidelli et al., 2009).

Exposure to artificial gravity provides protection against microgravity induced apoptosis and oxidative stress in retinal endothelial cells of rodents flown on ISS (Mao et al., 2018).

A profound rearrangement of the cytoskeleton network, dose-dependent increase of FAK phosphorylation, and Yes-associated protein 1 (YAP1) expression was found in dermal microvascular ECs exposed to hypergravity, suggesting improved motility and proangiogenic response. Transcriptome analysis showed changes in the expression of genes associated with cardiovascular homeostasis, NO production, angiogenesis, and inflammation (De Cesari et al., 2020). These results show that adaptation to hypergravity has opposite effects compared to microgravity on the same cell type, suggesting it as a potential physical countermeasure. Its real application is however far away.

In summary, the efficacy of physical countermeasures, alone or combined with other therapies, remains to be defined in cultured cells and in integrated and innovative tissue models in order to be effective and safe in a spaceflight arrangement.

## Conclusion and Perspectives

While the events and mechanisms controlling wound healing are well known and characterized, the pharmacological interventions to prevent or treat healing dysfunction are few and nowadays still under evaluation and validation on Earth. The data available in relation to unloading conditions document that the impaired wound healing results from the following mechanisms: 1) persistent inflammation with neutrophil infiltration (Dovi et al., 2003; Radek et al., 2008) and overall alteration of the inflammatory phase; 2) altered blood flow with more permeable vessels (McDonald et al., 1992), presumably linked to increased NO and VEGF levels (Shi et al., 2012; Dittrich et al., 2018); 3) an altered neovascularization that may result from impaired EC proliferation and migration in response to angiogenic factors, increased EC apoptosis, and altered gene expression and signalling pathways (Morbidelli et al., 2005; Radek et al., 2008; Li et al., 2018; Kasiviswanathan et al., 2020); 4) exposure to simulated microgravity also resulting in enhanced ROS production that may contribute to unloading-induced oxidative stress. The systemic oxidative status can be derived from radiation-induced immune system alterations especially relevant in long-duration space flight (Rizzo et al., 2012).

Considering the space environment and the critical issues characterizing long duration space travels (unloading, confinement, scarce hygiene, and radiations) is mandatory to further study angiogenesis and wound healing in space, to precisely define the target for therapeutic interventions and to validate efficient and safe countermeasures and treatment protocols. The combination of various stressors needs to be characterized. Recently, a paper by Mao et al. (2019) revealed the synergistic worsening effect of combined unloading and radiation exposure on oxidative stress and dysfunction of retinal ECs, events responsible for sight loss associated to space permanence.

Additionally, for a series of countermeasures, it is difficult to imagine their exploitation in a space environment since they require freshly isolated living cells or facilities and competences to extract/cultivate cells from the injured subject. In long duration missions, to deliver in time, ad hoc cells/tissues will be not possible and proangiogenic/regenerative countermeasures should be available on shelf/on board or rapidly achieved. Therefore, the effort of national and international space agencies goes in this direction and this review reflects the state of the art on the specific phenomenon of angiogenesis contribution on wound healing and the potentiality of developing effective and safe countermeasures. Up-to-date techniques are needed both for the study of the mechanisms of angiogenesis alterations in space environment and for the validation of countermeasures to improve wound healing. Examples are tissue engineering, cocultures, 3D multicellular structures, lab-on-chip approaches (Grimm et al., 2014; Ma et al., 2014; Huang et al., 2020), which are starting to substitute experimental animals due to ethical issues. Thorough comprehension of the molecular and biochemical mechanisms underlying cellular responses is coming from omics techniques and RNA sequence analysis of samples from simulated and real microgravity experiments (Ma et al., 2014; Mao et al., 2018; Cui et al., 2020; Kasiviswanathan et al., 2020). The expectations from these types of experiments are high.

Nevertheless, it is important to stress the concept that all the information obtained for space research can be exploited on the Earth for fragile (aged, diabetic) or bed-ridden patients, whose clinical characteristics are very similar to astronauts. A common feature that accompanies space travelers and aged/fragile patients is indeed endothelial dysfunction, which, among the others, is responsible for angiogenesis impairment and not efficient healing of wounds and ulcers.
